# Research protocol local ingredients-based supplementary food as an alternative to corn-soya blends plus for treating moderate acute malnutrition among children aged 6 to 59 months: a randomized controlled non-inferiority trial in Wolaita

**DOI:** 10.1186/s12889-019-8031-3

**Published:** 2019-12-16

**Authors:** Debritu Nane, Anne Hatløy, Elazar Tadesse, Bernt Lindtjørn

**Affiliations:** 1 Hawassa University, School of Public and Environmental Health, PO Box 5, Hawassa, Ethiopia; 2 Centre for International Health, University of Bergen, PO Box 7800, 5020 Bergen, Norway; 3 Fafo Institute for Labour and Social Research, PO Box 2947, 0608 Oslo, Norway; 4 Kotebe Metropolitan University, PO Box 31228, Addis Ababa, Ethiopia

**Keywords:** Moderate acute malnutrition, Local ingredients-based supplement, Corn-soya blend plus, Randomized controlled non-inferiority trial, Effectiveness

## Abstract

**Background:**

In Ethiopia, 12.5% of children below 5 years are wasted, and 9.7% are moderately wasted. The present strategy for the management of moderate acute malnutrition (MAM) is a supplementary feeding program; however, this is only provided to chronically food-insecure areas. This randomized controlled non-inferiority trial examines if Local ingredients-based supplement (LIBS) is as effective as corn-soya blends plus (CSB+) in treating moderate acute malnutrition among children aged 6–59 months.

**Methods:**

A randomized controlled non-inferiority trial will be conducted with moderately wasted children aged 6 to 59 months in Wolaita, Ethiopia. The calculated sample size is 324 (i.e. with 162 children in each of two arms, to be assigned by randomization). The daily ration will be: 100 g of LIBS plus 25.2 g of sugar with 8 ml oil in the intervention group, and 150 g of CSB+ with 16 ml of oil in the control group. These interventions will be provided for a maximum period of 12 weeks, with follow-up performed on a weekly basis. Data analysis will be done using SPSS and STATA software. Both intention-to-treat and per protocol analyses will be done. Hazard ratio and Kaplan-Meier (log rank) curves of survival analysis will be done to predict the probability of recovery rate. Logistic regression will be used to test for interactions between independent and dependent variables. Analysis of variances, t-tests, fisher’s exact test and chi-square tests will be used to assess baseline characteristics.

**Conclusions:**

This paper will introduce to the existing research locally available nutritious foods which have the potential to enhance recovery from moderate acute malnutrition and to reduce the burden of malnutrition. The perceptions of mothers on feeding children with local ingredient-based supplementary food to assist recovery from moderate acute malnutrition will be the focus of in a qualitative study to follow; this will provide a further contribution in an evolving area of research.

**Trial registration:**

Pan-African Clinical Trial Registration number: PACTR201809662822990, retrospectively registered on 11/09/2018.

## Background

Worldwide, acute malnutrition is a major contributor to deaths and disabilities in children, affecting about 51.5 million children below 5 years of age [1, 2]. Chronic food insecurity, poverty, poor feeding practice, limited household food availability and infections are considered possible reasons for developing acute malnutrition [3]. Acute malnutrition is associated with at least 12.6% child mortality, occurring among children aged below 5 years [2].

The Ethiopian Demographic and Health Survey of 2011 showed that about 10% of children aged below 5 years had acute malnutrition, and among these, 70% had MAM, defined by weight-for-height z-score (WHZ) between − 3 to − 2 Standard Deviations and/or mid-upper arm circumference (MUAC) of between ≥11.5 cm and < 12.5 cm, without bilateral pitting edema [4, 5]. Most malnutrition-related deaths occur in mildly or moderately malnourished children. In low- and middle-income countries, acute malnutrition is associated with about one in six deaths. Of these, 10.2% are due to MAM [6]. The death risk among moderately malnourished children is three-fold compared with that of non-malnourished children [1].

Children with MAM are susceptible to develop severe acute malnutrition (SAM) if they are not managed adequately. It is known that SAM has a high contribution to the global morbidity and mortality of young children. Therefore, children with MAM need to be treated with adequate nutrient-dense foods to prevent progressing to the life-threatening condition [2, 4].

In Ethiopia, the existing strategy for the management of MAM is a supplementary feeding program; however, this is restricted to districts where chronic food insecurity is present. In the areas not determined to be chronically food insecure, there are no food supplementation programs for MAM children. In these districts, the strategy to manage MAM children includes vitamin A supplementation, deworming and dietary counseling provided to the families [2]. The dietary counseling is provided to the families in these districts on the understanding that they have access to all foods required for child feeding, but lack knowledge on how best to use them [7].

In a study done in South-Western Ethiopia, without having a targeted nutrition-specific supplement to address MAM, only half of children aged 6 to 59 months recovered from MAM within 28 weeks of follow-up, with health and dietary counseling services alone. They were provided such services according to the national policy, but the recovery rate remained unsatisfactory. Even though these districts are named as food-secure areas, there are food-insecure households with acute moderately malnourished children suffering from high rates of deterioration and no improvement with the existing management. This is because household food insecurity is not determined by the food security level of districts [2].

Clinical strategies on the management of SAM have been present for many years. The implementation of these strategies has yielded positive outcomes. However, consistent study on the management of MAM is lagging behind, and much of the data is published on SAM, even if MAM is more prevalent and a gateway to developing SAM [8–10].

Children with MAM living in food-insecure areas are getting corn soya blend plus (CSB+) as a conventional supplement. Generally, CSB+ is given as a treatment to MAM children in developing countries since it has a good nutritional value for limited cost. However, there has been considerable argument in the international nutrition community concerning the nutritional suitability and competence for the treatment of MAM. CSB+ is believed by some to be a poor choice as a treatment since it does not contain important nutrients in acceptable amounts; it contains comparatively high amounts of anti-nutrient and fiber. In addition, it has a generally low fat level, and thus needs vegetable oil in order to provide the essential fatty acids and energy [11]. According to the report of different literature, the recovery rate after 8 to 16 weeks follow-up with CSB+ treatment is within the range of 67 to 76.8% [12–14].

The nutritional management of MAM have to normally be initiated on optimum usage of Local ingredient-based nutritious food [4]. Local ingredients based supplementary food are available, accessible, can be formulated at the household level and contain adequate amounts of the required nutrients [13].

In this paper, the new supplementary food referred to as Local ingredients based supplement (LIBS) made of locally available ingredients such as pumpkin seed, peanut, amaranth grain, flaxseed, and emmer wheat. Their portion designed to have the required amount of nutrients for the management of MAM among children aged 6 to 59 months.

In Ethiopia, Pumpkin cultivates widely and it is a seasonal crop that has been used for human food. Traditionally, consumers in Ethiopia using sun dried pumpkin fruit targeting at extended period storage for use during off season as part of food security plan [15]. Pumpkin is usually grows both in maize fields and vegetable gardens as well as in other more intensive agricultural systems [16]. Amaranth grain is a widely growing seed in Ethiopia [17]. It is mainly cultivated by people living in Southern Ethiopia as intercropped with sorghum and maize [18]. Emmer wheat is grown-up in marginal land in almost all districts of Ethiopia in both ‘Meher’ (June to September) and ‘Belg’(March to May) time of year [19]. Oilseed crops like peanut and flaxseed are widely cultivated in Ethiopia and consistently found in the local markets.

To confirm timely treatment of MAM and resolve the restricted distribution of supplement among beneficiaries, LIBS need to be developed. Besides this, adequate research has not been conducted in Ethiopia on Local ingredient-based supplementary food, although it has the potential to contribute to treating MAM. The information drawn from this study will be shared with the public, policymakers and academics, as locally available nutritious foods have the potential to enhance recovery from MAM and reduce the burden of malnutrition.

### Aim of the study

The objective of this study is to evaluate if LIBS is as effective as standard corn-soya blend plus in treating MAM among children aged 6 to 59 months.

### Hypothesis

In MAM children aged 6 to 59 months, intervention with LIBS will, within 12 weeks (with a 7% margin of non-inferiority), will not have an inferior recovery rate as children with MAM receiving CSB+.

## Methods

### Study design

A randomized, controlled, non-inferiority trial will be carried out to evaluate the efficacy of LIBS (the intervention), compared with conventional treatment which is CSB+ (a control), in treating MAM.

### Setting

This study will be conducted in Damot Pulassa district located in Wolaita zone. Like other districts of Wolaita, this district has the highest population densities of more than 700 persons per square kilometer [20]. The district characterized by having a fragmented farm and land ownership. The disparity between land and population balance has by far persisted the main contributing factor for the occurrence of endemic food insecurity [21]. Damot Pulassa district is classified as a maize and root crop livelihood zone because such crops are the main ones produced in the area. The district is serving by five health centers and 23 health posts. These health posts are led by health extension workers and deliver nutrition-linked services like nutrition education, screening for nutritional status of young children and nutritional management of the malnourished ones. The district was selected based on a consideration of the high level of food insecurity, high level of child malnutrition, good geographical location and access to transportation.

### Study population

The study population will be children with MAM, MUAC of ≥11.5 cm and < 12.5 cm without bilateral edema and/or with weight-for-height z-scores between − 3 to − 2 Standard Deviations [4, 5]. Mothers or caregivers of selected children will be the respondents.

### Inclusion criteria

- All children 6 to 59 months of age identified as MAM (MUAC of ≥11.5 cm and < 12.5 cm) [5].

### Exclusion criteria

- Children with SAM based on WHO 2009 child growth standards and/or children with bilateral pitting edema [22].
- Children with any illness or other medical complications that prevent them from safely consuming supplementary food.
- Children already participating in other interventions.

### Sample size calculation

The proportion of children who recover from MAM in an area where there is a supplementary feeding program (CSB+) is about 67% [12]. The sample size assumes that the expected percentage response in an experimental group is 60% and in a control group is 67%, with the non-inferiority criterion set to be an absolute value of 7%. To achieve 80% power to demonstrate non-inferiority, it is estimated that 162 subjects per group would be required, including a 10% withdrawal rate. This leads to a total required sample size of 324 subjects. The anticipated 10% dropout rate was used based on the observed dropout rate reported in two studies [12, 13]. For this sample size calculation, the PharmaSchool sample size calculator for non-inferiority trials was used.

### Recruitment of study participants and randomization

Data collectors with health extension workers will visit all households with children aged 6 to 59 months. They will assess for eligibility by measuring the children’s MUAC. If the child has a MUAC of < 12.5, data collectors will register and send the caregiver with the child to the actual screening site, where MUAC will be re-measured and weight and height or length will be taken. Children aged 24 to 59 months will be measured for weight and height, and children aged 6 to 23 months will be measured for weight and length. In addition to the values of MUAC (i.e. 11.5 cm to 12.5 cm), we will use the z-scores of weight for height or weight for length for recruitment (i.e. weight-for-height z-scores: between − 3 to − 2 Standard Deviations). If the child is identified as moderately malnourished according to the MUAC and not according to the values of weight-for- height/length z-scores, we will recruit them with their MUAC values. The screening procedure will be conducted by trained data collectors and facilitated by health extension workers using both MUAC and WHZ. Edematous malnutrition will also be evaluated using bilateral pitting edema criterion. The screening process will be continued until the sample size is met.

**Fig. 1:**
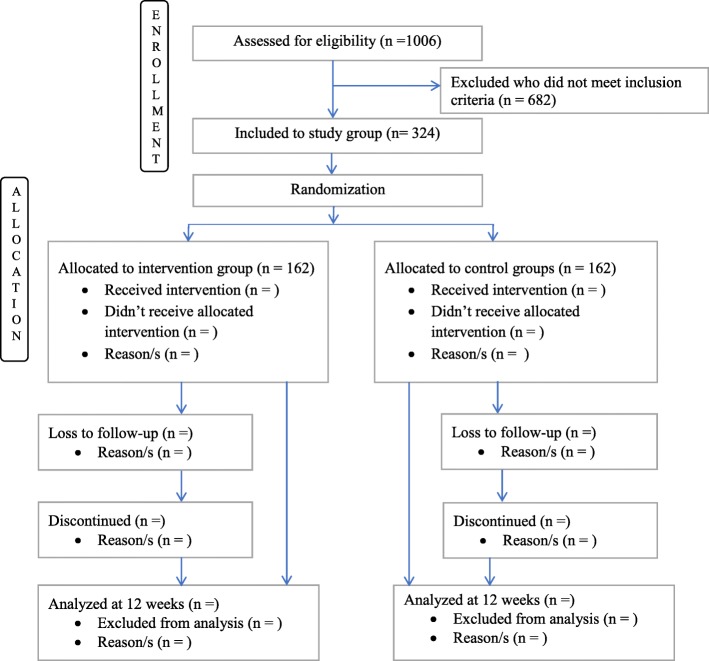
Trial profile. Assessed for eligibility = Children aged 6 to 59 months who assessed for moderate acute malnutrition.. Randomized = Children aged 6 to 59 months who assessed for moderate acute malnutrition, fulfilled the inclusion criteria, recruited and randomly allocated. Loss to follow-up = Children who are randomly allocated to intervention and control groups and stop to participate in the study at any stage of the study. Discontinued = Children who are randomly allocated to intervention and control groups and discontinued from the study at any stage of the study. Analyzed at 12 weeks = Children aged 6 to 59 months whose information analyzed at 12 weeks

Computer generated sequentially numbered randomization list that contained codes for children who meet enrolment criteria will be prepared by the research supervisor. These children will be randomized into intervention or control groups, using random allocation software. The allocation ratio will be 1:1 (Fig. 1).

Allocation of participants to the intervention or control group will be done by one of the research team who has no information about the participant identity. After allocation into two groups, the subjects further categorized by the investigator into sub-groups, with equal numbers of children based on their location/neighborhood to assign one food distributor to each group in which food distributor can easily reach the households with the selected child.

### Blinding

Caregivers, data collectors and food distributers will be blinded for the intervention. Both supplements will be packed with similar packs, and will be distributed and prepared in the same way. The sugar which will be distributed for the intervention group will be mixed with the flour before packing. Vegetable oil will be distributed for both intervention and control groups with the supplement and food distributer will be assigned to help caregivers in cooking the porridge with 16 ml of oil for CSB+ group and with 8 ml of oil for intervention groups. The data will be analyzed by an individual other than the assessor.

### Data collection

Selected caregivers of children with MAM aged 6 to 59 months will be interviewed for collection of baseline data such as socio-demographic and economic status, dietary habits, child’s age, breastfeeding practices and history of child and maternal illness. The child’s weight will be noted using a seca weight scale to the nearest 0.1 kg. The data collectors will be sure that the scale is placed on a flat, hard, even surface and will weigh with minimal clothing.

Height will be recorded to the nearest 0.1 cm using a seca height scale for children aged 24 to 59 months. For children aged 6 to 23 months, length will be measured to the nearest 0.1 cm using a locally made wooden measuring board. Before starting height measurement, the data collectors will ensure that the height board is on level ground and the child is without shoes; the collector will kneel in order to get to the level of the child and will encourage the caregiver to help. For length, data collectors will measure the child lying down, being sure that the length board is placed on a flat and stable surface.

MUAC will be documented by non-stretchable standard United Nations Children’s Fund (UNICEF) plastic tape measures. The measurement will be taken half-way between the acromion and the olecranon processes, with the measuring tape fitting comfortably, but without making a depression in the upper arm. Weekly assessment of anthropometric measurement using MUAC will be done by the data collectors for intervention as well as control groups. On a monthly basis, data collectors will again collect anthropometrics using the MUAC, height or length board and weight scale which are identical equipment to that used at baseline. At the same time, they will observe for bilateral pitting edema. Based on the follow-up measurement of anthropometry children who developed sever acute malnutrition will be referred to SAM clinic.

### Description of interventions and distribution of supplementary foods

After collecting baseline information, each child in the intervention group will be given local ingredient-based supplement of 100 g with 20 g of sugar. The ration will be distributed on a daily basis for 12 weeks. The LIBS is made from 30 g of pumpkin seed, 25 g of peanut grain, 20 g of amaranth grain, 15 g of flaxseed, 10 g of emmer wheat, 25.2 g of sugar and 8 ml of oil. This supplement yielded 698.5 kcal (22.6 g protein, 40.89 g fat and 60.05 g carbohydrate). Similarly, each child in the control group will be given the conventional supplement (CSB+) with the amount of 150 g flour/day with 16 ml of oil (763.98.4 kcal, 23.5 g protein and 25 g fat) on a daily basis for a period of 12 weeks. The subjects will be served with both supplements in the morning as a breakfast (Table 1).

**Table 1:** Nutrient composition of the supplementary foods

Nutrient	100 g of LIBS with 25.2 g of sugar plus 8 ml oil	150 g of CSB+ with 16 ml of oil
Energy (kcl)	698.5	763.98
Protein (gm)	22.6	20.25
Fat (gm)	40.89	30.76
Iron (mg)	8.1	6
Zn (mg)	5.6	7.5
Calcium (mg)	100	195
Phosphorous (mg)	470.55	300
Potassium (mg)	666.14	600
Magnesium (mg)	394.7	107.75
Sodium (mg)	84.6	41.25
Folic acid (μg)	49.4	90

Note: Nutrient values for the LIBS ration were calculated by using the USDA food composition database and NutriSurvey software. Nutrient values for the CSB+ was adapted from Amegovu KA, Ogwok P, Ochola S, Yiga P, Musalima HJ, Mutenyo E. Formulation of sorghum-peanut blend using linear programming for treatment of moderate acute malnutrition in Uganda. J Food Chem and Nutr. 2013; 1(2):67–77

Abbreviations: *LIBS* Local ingredients based supplement, *USDA* United States Department of Agriculture and *CSB+* Corn soy blend plus

According to the guidelines for selective feeding, all children 6–59 months of age will receive the same amount of food [23]. During the intervention period, the food distributers will visit households daily to assist the caregivers in the preparation of porridge and feeding, to advise, assess and resolve difficulties with feeding. Besides, they will check for and quantify the amount of supplement consumed by the children.

### Data quality management

Scheduled as well as unscheduled home visits and close follow-up to check feeding procedure will be made by the supervisors and investigator. If the child is a twin, an additional amount of supplementary food will be given to the caregiver to confirm that the enrolled child is provided with a full portion.

If two children with MAM are found in the same household, both children will receive food but only the randomly selected child will be enrolled in the study. If the recruited child is not at home during the time of follow-up, data collectors will revisit those households until they find the child. Sessions for standardization of anthropometric measurements will be carried out on a monthly basis. Monitoring of the food distribution process, feeding techniques and use of the provided food supplements will be carried out among randomly selected households on a twice-monthly basis. After data collection, the filled questionnaires will be stored in a secured place, and data entry will start. Double data entry and checking for consistencies will be carried out.

### Data analysis

All data collection sheets will be controlled by the field supervisors for completeness. EpiData version 3.1 will be used for all data entry. Two data clerks will enter data simultaneously to ensure data quality. Continuous data quality evaluation will be made through automatic consistency checks. Statistical significance is set at 5% for all analyses. Statistical analysis will be done using SPSS and STATA software for survival and hazard ratio. The probability of recovery rate will be predicted in Kaplan-Meier (log rank) curves of survival analysis. T-tests, analysis of variance (ANOVAs) and chi-square tests will be used to assess baseline characteristics. In accordance with recommendations for analyzing and reporting equivalence and non-inferiority trials, both intention-to-treat (ITT) and per protocol (PP) analyses will be done, and the 95% CI will be used to infer whichever variances are significant [24]. The ITT analyses will include all children enrolled in the study whereas the PP analyses will exclude children who lost to follow-up, refused and transferred out of the management programme but include children discharged as cured, died or non-cured.

Logistic regression will be used to test for interactions between the recovery rate and other variables. Mean recovery time and median recovery time, mean differences of MUAC and mean difference between WHZ scores will be computed to describe the magnitude of the difference between the two groups.

### Outcome variables

The primary outcome measure of this study will be recovery rate. ‘Recovered’ will be defined as when a child attains a MUAC ≥12.5 cm and WHZ scores ≥ − 2, without bipedal edema. The secondary outcomes are the mean recovery time and average weight gain. Children who developed SAM during the study and/or persisted as moderately malnourished at the end of the 3-month follow-up will be considered to have failed the management for MAM.

### Participant timeline

**Fig. 2:**
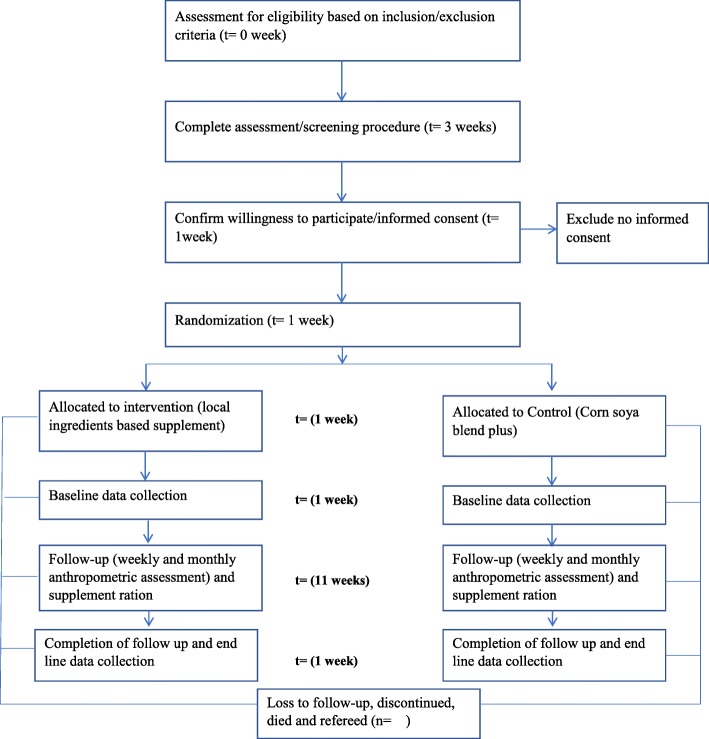
Participants time line. Assessed for eligibility = Children aged 6 to 59 months who assessed for moderate acute malnutrition based on inclusion/exclusion criteria. Complete assessment = Completion of screening for moderate acute malnutrition. Confirm willingness to participate = be sure for the willingness of care givers of the subject to be included in the study / informed consent. Randomization = Process of randomizing the subjects/ children aged 6 to 59 months who assessed and claimed as moderately malnourished. Base line data collection = collection of data before starting food ration. Follow-up = Weekly as well as monthly anthropometric assessment and daily food ration. End line data collection = Collection of data after completion of the follow up. t = indicates time that the processes have done/completed

Children aged 6 to 59 months will be assessed for MAM based on inclusion and exclusion criteria using MUAC and weight-for-height/length z-scores. The screening procedure will end within 3 weeks. Consenting of the eligible respondents (mothers of children aged 6 to 59 months) and randomization of the eligible subjects will take place 1 week after screening completed during which the subjects will be allocated into intervention and control groups, along with baseline data collection. Follow-up interviews with the anthropometric assessment will be done weekly and monthly basis after the allocation date and completed within 11 weeks. End-line data collection will take place 1 week after ending a follow-up interview (Fig. 2).

### Training

Data collectors will receive training about the objectives of the study, data collection systems, interview techniques, anthropometric measurements, feeding procedures and field procedures prior to the data collection. Each question found in the questionnaire will be discussed during training, in which trainers will develop common understandings.

Standardization of anthropometric measures will be maintained. If it is possible, the same assessors will be used for all assessments.

### Pretesting

Pretesting of the questionnaire will be conducted in places away from where the actual study will be conducted. For the pretesting, 16 pairs of children and caregivers, which is 5% of the total sample size, will be interviewed to check the accuracy of questions and to decide on the length of interviews. During pretesting, consistency in the interpretation of questions will be checked to identify any unclear items. After the evaluation of instruments, all proposed revisions will be done before administering in the actual study.

### Harms

The food ingredients used for formulating the supplement will be foods which are already being used in these communities and are safe for consumption. The participants will get supplementary food at no cost as the treatment for their malnutrition status. There might be minimal risk of allergic reaction. There might be risk while making interactions and spending time through the process.

### Auditing

The study is open for auditing by any concerned organization, either government or non-government.

### Protocol amendment

Communication will be done regarding the protocol modification with those important stakeholders (IRB/IEC and Sponsor).

### Dissemination of the result

The result of this study will be disseminated through Internal Seminars, Conference presentations using outreach and public engagement events. Regular reporting and communication to stakeholders will be done. It will also disseminate through Publications.

### Potential benefits

This project will be carried out in the kebeles (neighborhoods) found in Wolaita zone where there is no provision of a supplementary feeding program. The children participating in this study will get supplementary food for their malnutrition. The intervention will enhance recovery from MAM, and those recovered from MAM will be discharged and rejoin the community.

Children who may develop serious allergic reactions like difficulty in breathing, dizziness, weakness or any unusual signs will be referred for treatment. Such participants will be excluded from the trial. Children who are temporarily sick or do not feel well will be sent to the health facility for the treatment, and their measurements will be rescheduled for other days.

### SPIRIT guidelines

The study protocol followed the SPIRIT guidelines for randomized controlled trials.

## Discussion

MAM is a state that needs to be managed before it progresses to the life-threatening condition which is SAM. It is known that SAM has a high contribution to the global morbidity and mortality of young children [2]. Children with MAM need to achieve catch-up growth in weight and height, and they must be able to fight against infection and disease. That is why, currently, specific nutrient recommendations have recently been established for children with MAM living in developing countries [10].

The 2012 WHO technical note on supplementary foods for treating MAM in children aged 6 to 59 months recommends the delivery of locally available, nutritious foods to enhance nutritional status and prevent SAM [4]. It is important to evaluate the effect of feeding acute moderate malnourished children with local ingredient-based supplementary food. The food supplement referred in this paper is LIBS made from locally available ingredients either cultivated locally or found in the local market. If this study demonstrates positive results, it will provide a direction to the public, policymakers and academics, as locally available nutritious foods have the potential to enhance recovery from MAM and reduce the burden of malnutrition. The current study intends to determine the recovery rate from MAM by examining baseline and end-line measurements of MUAC and WHZ scores of the subjects. It will also evaluate the average weight gain and time of recovery.

## Trial status

The trial was registered with the Pan-African Clinical Trial Registry on 11/09/2018 and the trial number is PACTR201809662822990.

## Data Availability

The datasets used and/or analyzed during the current study will be available from the corresponding author, on reasonable request.

## Abbreviations

ANOVA: Analysis of Variance
CSB +: Corn-Soy Blended Flour Plus.
ITT: Intention-to-Treat
LIBS: Local Ingredients Based Supplement
MAM: Moderate Acute Malnutrition
MUAC: Mid-Upper Arm Circumference
PACTR: Pan-African Clinical Trial Registry
PP: Per Protocol
SAM: Severe Acute Malnutrition
SFP: Supplementary Feeding Program
UNICEF: United Nations Children’s Fund
WHO: World Health Organization
WHZ: Weight-for-Height Z-score
